# Status and Factors Associated With Patient Safety Culture in Traditional Chinese Medicine Institutions: A Cross‐Sectional Study

**DOI:** 10.1002/hcs2.70029

**Published:** 2025-08-12

**Authors:** Liujin Zhang, Chen Wei, Yuan Le, Fuqiang Chen, Tao Yang, Zhiwei Leng

**Affiliations:** ^1^ Center for Health Policy and Health Economics, National Infrastructures for Translational Medicine, Institute of Clinical Medicine, Peking Union Medical College Hospital Chinese Academy of Medical Sciences and Peking Union Medical College Beijing China; ^2^ School of Public Health China Medical University Shenyang China; ^3^ General Office of the CPC Committee, Peking Union Medical College Hospital Chinese Academy of Medical Sciences and Peking Union Medical College Beijing China; ^4^ Peking Union Medical College Education Foundation Chinese Academy of Medical Sciences and Peking Union Medical College Beijing China

**Keywords:** associated factors, current status, patient safety culture, traditional Chinese medicine institutions

## Abstract

**Background:**

Strengthening the patient safety culture is essential for improving healthcare service quality and ensuring patient safety. The study's aim was to investigate the current status of patient safety culture in traditional Chinese medicine institutions and its associated factors to provide scientific recommendations for improvement.

**Methods:**

This cross‐sectional study was conducted October 14–18, 2024. Four traditional Chinese medicine institutions in Zhejiang Province, China, were selected using convenience sampling, and the expected sample size was 544 participants. Data were collected using the Hospital Survey on Patient Safety Culture questionnaire, which comprises two sections: individual characteristics and patient safety culture. The latter comprises 10 dimensions with 32 items rated on a five‐point Likert scale. Dimension scores were calculated as the mean of corresponding item scores, and the overall score as the mean of all dimension scores. Descriptive statistics, *t*‐tests, analysis of variance, and multiple linear regression were used to analyze current status and associated factors of patient safety culture.

**Results:**

A total of 522 valid questionnaires were collected. The average patient safety culture score was 3.92. Among the dimensions, “F Communication About Error” received the highest score and “H Reporting Patient Safety Events” the lowest score. Age, monthly income, hospital grade, professional title, and weekly working hours were identified as independent factors associated with patient safety culture.

**Conclusion:**

The overall patient safety culture in the traditional Chinese medicine institutions of Zhejiang Province is relatively high but is affected by multiple factors. Further efforts are needed to implement diverse strategies aimed at strengthening patient safety culture within traditional Chinese medicine institutions.

## Graphical abstract

This study investigated the status and determinants of patient safety culture in traditional Chinese medicine institutions in Zhejiang Province. Results showed a relatively higher patient safety culture, with “Communication About Error” scoring highest and “Reporting Patient Safety Events” lowest. Key influencing factors include age, income, hospital grade, professional title, and working hours.

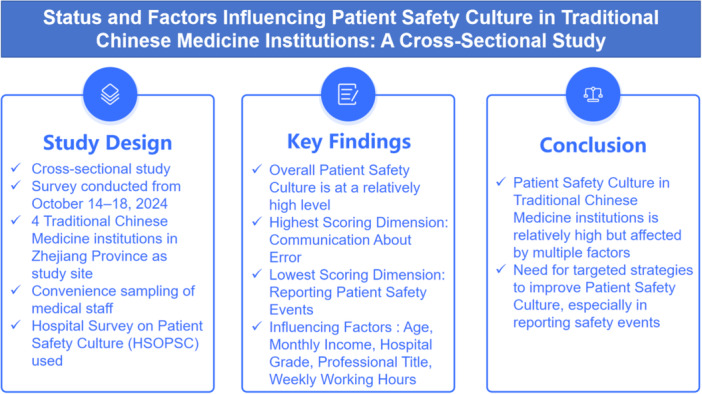

**Abbreviations**
HSOPSC: Hospital Survey on Patient Safety Culture
PSC: patient safety culture
TCM: traditional Chinese medicine

## 1 Background

Patient safety is central to healthcare management and affects the lives and health of populations [1]. The World Health Organization has defined patient safety as “the absence of preventable harm to patients and reduction of risk of unnecessary harm associated with health care to an acceptable minimum,” and considers “do no harm” to be the fundamental principle of healthcare services [2]. In 2021, the 74th World Health Assembly developed the first “Global Patient Safety Action Plan 2021–2030,” which aims to eliminate avoidable harm in healthcare [3]. In 2023, China released the “Patient Safety Special Action Plan,” which is designed to promptly remove various risks in the medical process and hospital environment, minimize unnecessary harm to patients during hospital stays, and ensure patient safety [4]. Thus, both China and the international community are committed to fostering a good patient safety environment to safeguard patients.

Patient safety culture (PSC) refers to the shared attitudes, beliefs, values, and behaviors among healthcare facility staff and is aimed at ensuring patient safety [5]. Strengthening the PSC can increase healthcare professionals' awareness of safety issues, reduce medical errors, and protect the health and safety of patients [6]. To this end, the National Health Commission of China issued the “Patient Safety Special Action Plan” in 2023, which mandated the integration of PSC into the overall development goals of hospitals and leveraged the guiding role of cultural development in patient safety management [4]. The cultivation of PSC is essential for safeguarding patients' health rights, ensuring patient safety, and improving the level of patient safety management in healthcare institutions.

Traditional Chinese medicine (TCM) is an important part of China's rich traditional culture, and has effectively maintained patient health and improved the health standards of the population in clinical practice. However, TCM institutions face growing challenges in patient safety management, highlighting the urgent need to strengthen PSC. The lack of standardized diagnostic and therapeutic protocols increases clinical variability and hampers communication and error reporting. Additionally, 2024 reports of adverse events associated with TCM showed an increase, with serious cases accounting for 8.3%, an increase of 0.7 percentage points compared with 2023 [7]. These challenges highlight the importance of strengthening PSC in TCM institutions to ensure both patient protection and the sustainable, high‐quality development of the institutions.

Although improving PSC in TCM institutions is very important, empirical evidence of the current status of PSC and factors associated with it remains limited. Previous studies have mainly focused on Western medicine settings [8, 9, 10], exploring the current status and related factors of PSC and offering evidence‐based strategies to improve PSC in those institutions. However, the PSC in TCM institutions may differ from that in Western medical institutions because of differences in theoretical foundations, treatment philosophies, and methods between Western and Chinese medicine. For example, TCM is based on holistic concepts and personalized treatment using methods like herbal medicine, acupuncture, and massage, whereas Western medicine focuses on specific local treatment using methods like pharmaceuticals and surgery.

This study focused on TCM institutions, with the aim of understanding the current status of PSC in TCM institutions and its associated factors, as well as providing scientific suggestions for improving PSC in TCM institutions.

## 2 Methods

### 2.1 Study Design

This was a cross‐sectional survey. The aim was to assess the current status of PSC in TCM institutions and identify related factors to provide an evidence‐based foundation for strengthening PSC in these settings.

### 2.2 Survey Instrument

The Hospital Survey on Patient Safety Culture (HSOPSC) scale developed by the US Agency for Healthcare Research and Quality was used to measure PSC in TCM institutions. The HSOPSC has been widely used to measure PSC and has demonstrated good reliability and validity in practice [11, 12, 13]. The scale consists of 32 items on 10 dimensions, including 13 negative items (A3, A5, A6, A7, A9, A11, A13, A14, B2, C7, F3, F4, F5). Each item has six response options: “strongly disagree,” “disagree,” “neutral,” “agree,” “strongly agree,” and “not applicable/do not know.”

A literature review was conducted to determine relevant respondent characteristics. Data were collected on demographic characteristics (e.g., gender, age), work‐related characteristics (e.g., hospital grade, years of service at current hospital), and PSC‐related characteristics (e.g., direct contact with patients, familiarity with PSC). The effect of these characteristics on the PSC of medical institutions was explored. Supporting Information S1 provides details of the survey instrument.

### 2.3 Data Collection

According to one rule of thumb, the required sample size for survey research is 5–10 times the number of questionnaire items. As the questionnaire contained 49 items, the required sample size was 245–490. Assuming an effective response rate of 90%, the adjusted required sample size was 272–544. A larger sample size helps to reduce sampling error and improves the reliability of the results. Therefore, it was planned to collect 544 completed questionnaires. The inclusion criterion was full‐time medical staff employed at the selected institutions. Quality control measures included restricting each account to a single submission to avoid duplicate responses and excluding questionnaires completed in under 10 min as invalid. Using convenience sampling, four TCM institutions in Zhejiang Province, China, were selected as study sites: Zhejiang Provincial TCM Hospital, Fuyang District TCM Hospital in Hangzhou, Jiande TCM Hospital, and Hangzhou Tuina Hospital. From October 14–18, 2024, a questionnaire survey was conducted among healthcare professionals at these institutions using the online survey platform Questionnaire Star. A total of 582 questionnaires were collected, of which 60 were excluded owing to insufficient completion time, resulting in 522 valid questionnaires and an effective response rate of 90%.

### 2.4 Data Analysis

In this study, each HSOPSC item was measured using a five‐point Likert‐type scale ranging from 1 (“strongly disagree”) to 5 (“strongly agree”). Negatively worded items were reverse‐coded before analysis, and responses marked as “not applicable/do not know” were treated as missing values. Dimension scores were calculated as the average of all item scores on each dimension, and the overall PSC score was derived from the mean of all dimension scores.

Data analysis was conducted using STATA 16.0. First, a descriptive analysis of the respondents' characteristics was conducted. Second, a descriptive analysis was performed on the respondents' PSC scores and positive response rates; the positive response rate was defined as the number of positive responses divided by the total number of responses. A positive response was defined as selecting “strongly agree/always” or “agree/most of the time” for a given item. A positive response rate ≥ 75% was considered excellent, while a rate < 50% was considered to indicate areas needing improvement. Third, *t*‐tests and analysis of variance were used to test for differences in the PSC of respondents with different characteristics. Finally, multivariate regression was used to explore the independent associated factors of PSC.

## 3 Results

### 3.1 Description of Respondent Characteristics

Table 1 summarizes the demographic characteristics of the 522 respondents. Most were female (95.79%), aged 31–45 years (49.62%), married (67.62%), and held a bachelor's degree or higher (82.57%). Most respondents (35.44%) had a monthly income of 5001–7000 CNY.

**Table 1:** General demographic characteristics of respondents (*n* = 522).

Characteristics	The number of respondents (*n*)	Proportion of respondents (%)
Gender		
Male	22	4.21
Female	500	95.79
Age (Years)		
≤ 30	209	40.04
31–45	259	49.62
> 45	54	10.34
Education Level		
Associate Degree or Below	91	17.43
Bachelor's Degree or Above	431	82.57
Marriage		
Unmarried*	169	32.38
Married	353	67.62
Monthly Income (After Tax, CNY**)		
≤ 5000	173	33.14
5000–7000	185	35.44
>7000	164	31.42

Abbreviation: CNY, Chinese yuan.

* Including Never Married, Divorced, and Widowed.

Table 2 summarizes the work‐related characteristics of the 522 respondents. Most were employed at tertiary hospitals (71.26%), with 54.21% having worked at their current hospital for ≤ 10 years. Senior titles were the least common (10.73%). A total of 63.22% of respondents were officially employed; internal medicine was the largest specialty (40.23%), and 56.51% worked 40–50 h weekly. Additionally, 66.48% worked weekly night shifts and 45.21% mentored students.

**Table 2:** Work‐related characteristics of respondents (*n* = 522).

Characteristics	The number of respondents (*n*)	Proportion of respondents (%)
Hospital grade		
Tertiary hospital	372	71.26
Secondary hospital	150	28.74
Years of service at current hospital		
≤ 10	283	54.21
10–15	107	20.50
> 15	132	25.29
Professional title		
Junior level or below	280	53.64
Intermediate level	186	35.63
Senior level	56	10.73
Employment type		
Officially employed	330	63.22
Contract‐based	117	22.41
Other	75	14.37
Department		
Internal medicine	210	40.23
Surgery	130	24.90
Others	182	34.87
Weekly working hours (hours)		
≤ 40	140	26.82
40–50	295	56.51
> 50	87	16.67
Night shifts per week		
No	175	33.52
Yes	347	66.48
Whether to teach students		
No	286	54.79
Yes	236	45.21

Table 3 summarizes the PSC‐related characteristics of the 522 respondents. Most (95.59%) reported direct patient contact, 7.66% indicated that patient safety incidents had occurred in their department, 77.39% were familiar with PSC, and 7.28% had not received PSC training in the last year.

**Table 3:** PSC‐related characteristics of respondents (*n* = 522).

Characteristics	The number of respondents (*n*)	Proportion of respondents (%)
Direct contact with patients		
No	23	4.41
Yes	499	95.59
Occurrence of PSC in the Department		
No	482	92.34
Yes	40	7.66
Familiarity with PSC		
Unfamiliar	21	4.02
Moderately familiar	97	18.58
Familiar	404	77.39
Participation in patient safety training in the past year		
No	38	7.28
Yes	484	92.72

Abbreviation: PSC, patient safety culture.

### 3.2 Current Status of Respondents' PSC

Table 4 shows the respondents' PSC scores and positive response rates. The overall average score was 3.915, with the mean score on all dimensions was above 3. Scores were lowest for “H Reporting Patient Safety Events” and were highest for “F Communication About Error.” The overall positive response rate was 81%, and exceeded 75% for all dimensions except “B Staffing and Work Pace” (70%) and “H Reporting Patient Safety Events” (40%).

**Table 4:** PSC scores and positive response rates of respondents.

Dimensions	Scores (x̄ ± s)	Positive response rate
A Teamwork (Teamwork)	4.043 ± 0.572	84.744
B Staffing and work pace (Staffing)	3.725 ± 0.721	69.703
C Organizational learning—continuous improvement (Learning)	4.046 ± 0.524	86.844
D Response to error (Error response)	3.852 ± 0.632	76.830
E Supervisor, manager, or clinical leader support for patient safety (Leader support)	4.044 ± 0.545	87.516
F Communication about error (Error communication)	4.250 ± 0.497	97.249
G Communication openness	3.958 ± 0.532	85.198
H Reporting patient safety events (Event reporting)	3.126 ± 1.029	40.160
I Hospital management support for patient safety (Management support)	4.066 ± 0.567	88.789
J Handoffs and information exchange (Handoffs)	4.011 ± 0.647	85.539
Total	3.915 ± 0.428	81.325

Abbreviation: PSC, patient safety culture.

### 3.3 Differences in PSC Among Respondents With Different Characteristics

Table 5 shows the comparison of PSC across demographic groups. Female respondents scored significantly higher than male respondents on the dimensions “Teamwork,” “Staffing,” “Leader Support,” “Error Communication,” “Management Support,” and overall PSC. Respondents aged ≤ 30 years outperformed those aged ≥ 45 years on the dimensions “Leader Support” and “Management Support.” Those with a bachelor's degree or higher scored significantly higher than respondents with an associate degree or below on the dimensions “Teamwork,” “Leader Support,” “Error Communication,” “Communication Openness,” and overall PSC. Compared with respondents earning ≤ 5,000 CNY, those earning 5,001–7,000 CNY scored higher on “Teamwork,” “Error Communication,” “Communication Openness,” “Management Support,” “Handoffs,” and overall PSC. Respondents earning > 7,000 CNY scored higher on “Teamwork,” “Staffing,” “Error Response,” “Leader Support,” “Error Communication,” “Handoffs,” and overall PSC. Marital status had no significant effect on PSC scores.

**Table 5:** Comparison of PSC by demographic characteristics.

Variables	Teamwork	Staffing	Learning	Error response	Leader support	Error communication	Communication openness	Event reporting	Management support	Handoffs	Total
Gender											
Male	3.803 ± 0.570	3.216 ± 0.780	3.894 ± 0.487	3.636 ± 0.591	3.689 ± 0.535	3.985 ± 0.418	3.778 ± 0.495	3.075 ± 0.963	3.773 ± 0.662	3.909 ± 0.593	3.680 ± 0.462
Female	4.054 ± 0.571	3.747 ± 0.711	4.053 ± 0.525	3.862 ± 0.632	4.060 ± 0.541	4.261 ± 0.497	3.966 ± 0.533	3.129 ± 1.033	4.079 ± 0.559	4.015 ± 0.650	3.925 ± 0.424
* t*	−2.016*	−3.417***	−1.390	−1.641	−3.148**	−2.570*	−1.590	−0.228	−2.492*	−0.751	−2.647**
Age											
≤ 30^a^	4.033 ± 0.576	3.754 ± 0.753	4.067 ± 0.556	3.871 ± 0.638	4.107 ± 0.563	4.287 ± 0.516	3.997 ± 0.580	3.117 ± 0.990	4.135 ± 0.569	4.013 ± 0.654	3.942 ± 0.439
31–45^b^	4.055 ± 0.566	3.734 ± 0.713	4.039 ± 0.515	3.852 ± 0.649	4.034 ± 0.537	4.248 ± 0.492	3.943 ± 0.510	3.114 ± 1.073	4.045 ± 0.564	4.025 ± 0.666	3.910 ± 0.428
>45^c^	4.022 ± 0.599	3.571 ± 0.620	3.997 ± 0.440	3.778 ± 0.513	3.852 ± 0.470	4.111 ± 0.420	3.884 ± 0.434	3.230 ± 0.965	3.901 ± 0.540	3.931 ± 0.516	3.833 ± 0.378
* F*	0.126	1.417	0.427	0.470	4.859**	2.714	1.342	0.280	4.052*	0.468	1.401
Pairwise comparison					a > c**				a > c*		
Education level											
Associate degree or below	3.929 ± 0.582	3.683 ± 0.842	3.978 ± 0.528	3.751 ± 0.607	3.894 ± 0.560	4.114 ± 0.485	3.826 ± 0.580	3.029 ± 0.975	3.995 ± 0.592	3.912 ± 0.674	3.814 ± 0.441
Bachelor's degree or above	4.067 ± 0.568	3.734 ± 0.694	4.060 ± 0.523	3.873 ± 0.635	4.076 ± 0.537	4.278 ± 0.495	3.986 ± 0.518	3.147 ± 1.040	4.081 ± 0.561	4.031 ± 0.640	3.936 ± 0.423
* t*	−2.108*	−0.535	−1.360	−1.676	−2.922**	−2.937**	−2.627**	−0.974	−1.324	**−**1.601	−2.475*
Marriage											
Unmarried	4.034 ± 0.565	3.751 ± 0.701	4.040 ± 0.549	3.878 ± 0.584	4.064 ± 0.524	4.249 ± 0.514	3.975 ± 0.559	3.065 ± 1.064	4.086 ± 0.530	3.980 ± 0.632	3.917 ± 0.422
Married	4.048 ± 0.577	3.712 ± 0.732	4.048 ± 0.513	3.840 ± 0.654	4.035 ± 0.555	4.250 ± 0.489	3.951 ± 0.520	3.155 ± 1.013	4.056 ± 0.584	4.025 ± 0.654	3.914 ± 0.432
* t*	**−**0.264	0.584	**−**0.163	0.642	0.572	**−**0.037	0.482	**−**0.915	0.555	**−**0.740	0.085
Monthly income											
① ≤ 5000^a^	3.923 ± 0.580	3.605 ± 0.773	3.965 ± 0.550	3.745 ± 0.625	3.948 ± 0.544	4.148 ± 0.476	3.871 ± 0.550	3.134 ± 1.038	3.973 ± 0.567	3.841 ± 0.689	3.819 ± 0.443
② 5001–7000^b^	4.100 ± 0.576	3.769 ± 0.695	4.084 ± 0.493	3.894 ± 0.625	4.065 ± 0.549	4.306 ± 0.543	4.027 ± 0.557	3.149 ± 1.055	4.121 ± 0.562	4.077 ± 0.628	3.962 ± 0.432
③ > 7000^c^	4.106 ± 0.544	3.801 ± 0.681	4.087 ± 0.525	3.918 ± 0.635	4.123 ± 0.529	4.293 ± 0.447	3.973 ± 0.472	3.093 ± 0.996	4.102 ± 0.563	4.115 ± 0.588	3.963 ± 0.391
* F*	5.814**	3.689*	3.072*	3.792*	4.604*	5.674**	3.937*	0.132	3.543*	9.251***	6.591**
Pairwise comparison	b > a* c > a*	c > a*		c > a*	c > a**	b > a** c > a**	b > a*		b > a*	b > a** c > a***	b > a** c > a**

Abbreviation: PSC, patient safety culture.

* *p* < 0.05

** *p* < 0.01

*** *p* < 0.001.

Table 6 shows the comparison of PSC across work‐related characteristics. Respondents from tertiary hospitals scored significantly higher than those from secondary hospitals on the dimensions “Teamwork,” “Staffing,” “Learning,” “Error Response,” “Leader Support,” “Error Communication,” “Communication Openness,” “Management Support,” “Handoffs,” and overall PSC. No significant differences were found based on length of service at the current hospital. Senior professionals scored higher on the “Error Response” dimension than those with intermediate or lower titles. Contract‐based staff (excluding officially employed staff) outperformed others on the dimension “Event Reporting.” Surgical department respondents scored higher on the “Teamwork” and “Handoffs” dimensions than those in internal medicine, while respondents from other departments scored higher than those in internal medicine on the “Teamwork” and “Error Communication” dimensions. Those working ≤ 40 h per week scored higher on the dimensions “Staffing,” “Error Response,” “Leader Support,” “Communication Openness,” “Handoffs,” and overall PSC than those working > 50 h, while respondents working 40–50 h scored higher on the dimensions “Staffing” and “Communication Openness” than those working > 50 h. Night shift frequency and teaching responsibilities had no significant effect on PSC.

**Table 6:** Comparison of PSC scores by work‐related characteristics.

Variables	Teamwork	Staffing	Learning	Error response	Leader support	Error communication	Communication openness	Event reporting	Management support	Handoffs	Total
Hospital grade											
Tertiary hospital	4.077 ± 0.579	3.788 ± 0.728	4.084 ± 0.539	3.899 ± 0.661	4.110 ± 0.553	4.295 ± 0.524	3.993 ± 0.567	3.145 ± 1.057	4.124 ± 0.579	4.086 ± 0.635	3.962 ± 0.438
Secondary hospital	3.960 ± 0.547	3.568 ± 0.681	3.951 ± 0.474	3.736 ± 0.536	3.881 ± 0.489	4.138 ± 0.400	3.872 ± 0.422	3.078 ± 0.955	3.923 ± 0.509	3.823 ± 0.640	3.798 ± 0.380
*t*	2.114*	3.178**	2.787**	2.684**	4.662***	3.696***	2.376*	0.658	3.903***	4.252***	4.273***
Years of service at current hospital											
≤ 10^a^	4.042 ± 0.579	3.719 ± 0.739	4.046 ± 0.545	3.841 ± 0.634	4.061 ± 0.561	4.260 ± 0.531	3.982 ± 0.565	3.137 ± 1.018	4.089 ± 0.583	4.021 ± 0.662	3.923 ± 0.436
10–15^b^	4.047 ± 0.605	3.813 ± 0.728	4.053 ± 0.530	3.873 ± 0.664	4.053 ± 0.510	4.234 ± 0.467	3.931 ± 0.501	3.090 ± 1.065	4.104 ± 0.535	4.022 ± 0.637	3.922 ± 0.429
> 15^c^	4.042 ± 0.534	3.665 ± 0.673	4.040 ± 0.474	3.859 ± 0.603	4.003 ± 0.541	4.240 ± 0.444	3.930 ± 0.484	3.134 ± 1.030	3.985 ± 0.551	3.978 ± 0.626	3.892 ± 0.411
*F*	0.003	1.263	0.019	0.107	0.528	0.149	0.602	0.081	1.829	0.216	0.247
Professional title											
Junior level or below^a^	4.026 ± 0.576	3.728 ± 0.752	4.040 ± 0.542	3.832 ± 0.627	4.054 ± 0.546	4.248 ± 0.489	3.956 ± 0.543	3.116 ± 1.030	4.077 ± 0.574	4.013 ± 0.647	3.912 ± 0.435
Intermediate level^b^	4.032 ± 0.565	3.715 ± 0.680	4.013 ± 0.502	3.820 ± 0.657	4.030 ± 0.529	4.237 ± 0.513	3.954 ± 0.515	3.080 ± 1.055	4.036 ± 0.547	3.972 ± 0.652	3.890 ± 0.410
Senior level^c^	4.164 ± 0.572	3.741 ± 0.710	4.185 ± 0.493	4.058 ± 0.533	4.045 ± 0.602	4.304 ± 0.485	3.987 ± 0.542	3.340 ± 0.919	4.107 ± 0.597	4.127 ± 0.624	4.013 ± 0.445
*F*	1.401	0.034	2.331	3.385*	0.113	0.396	0.088	1.34	0.462	1.225	1.814
Pairwise comparison				c > a* c > b*							
Employment type											
① Officially employed^a^	4.033 ± 0.570	3.695 ± 0.698	4.061 ± 0.516	3.852 ± 0.668	4.054 ± 0.542	4.277 ± 0.491	3.991 ± 0.520	3.139 ± 1.066	4.052 ± 0.580	4.022 ± 0.664	3.920 ± 0.436
② Contract‐based^b^	4.128 ± 0.570	3.811 ± 0.770	4.072 ± 0.540	3.915 ± 0.580	4.041 ± 0.585	4.251 ± 0.487	3.949 ± 0.564	3.254 ± 0.989	4.101 ± 0.592	4.006 ± 0.642	3.956 ± 0.432
③ Other^c^	3.956 ± 0.578	3.721 ± 0.741	3.940 ± 0.530	3.757 ± 0.530	4.007 ± 0.498	4.129 ± 0.522	3.830 ± 0.522	2.866 ± 0.874	4.073 ± 0.461	3.969 ± 0.586	3.830 ± 0.379
*F*	2.234	1.107	1.81	1.881	0.233	2.728	2.8	3.189*	0.316	0.208	2.035
Pairwise comparison								b > c*			
Department											
Internal medicine^a^	3.908 ± 0.568	3.692 ± 0.743	3.993 ± 0.530	3.801 ± 0.606	4.015 ± 0.551	4.183 ± 0.537	3.919 ± 0.533	3.173 ± 1.016	4.033 ± 0.565	3.951 ± 0.667	3.867 ± 0.416
Surgery^b^	4.153 ± 0.549	3.834 ± 0.634	4.112 ± 0.516	3.917 ± 0.650	4.072 ± 0.520	4.279 ± 0.471	3.941 ± 0.540	3.109 ± 1.059	4.145 ± 0.512	4.132 ± 0.594	3.971 ± 0.391
Others^c^	4.121 ± 0.565	3.685 ± 0.750	4.060 ± 0.520	3.864 ± 0.646	4.059 ± 0.557	4.306 ± 0.458	4.016 ± 0.524	3.082 ± 1.026	4.048 ± 0.602	3.994 ± 0.652	3.930 ± 0.462
*F*	10.267***	1.97	2.17	1.4	0.529	3.349*	1.701	0.386	1.72	3.250*	2.54
Pairwise comparison	b > a*** c > a**					c > a*				b > a*	
Weekly working hours											
≤ 40^a^	4.105 ± 0.570	3.865 ± 0.771	4.122 ± 0.513	3.924 ± 0.614	4.132 ± 0.532	4.314 ± 0.466	4.066 ± 0.486	3.083 ± 1.105	4.133 ± 0.538	4.098 ± 0.680	3.990 ± 0.443
40–50^b^	4.041 ± 0.586	3.726 ± 0.707	4.020 ± 0.531	3.861 ± 0.646	4.039 ± 0.547	4.253 ± 0.477	3.956 ± 0.551	3.146 ± 1.007	4.062 ± 0.541	4.019 ± 0.604	3.914 ± 0.421
> 50^c^	3.950 ± 0.520	3.497 ± 0.630	4.011 ± 0.512	3.708 ± 0.593	3.921 ± 0.540	4.134 ± 0.587	3.794 ± 0.502	3.129 ± 0.991	3.969 ± 0.677	3.841 ± 0.707	3.797 ± 0.403
*F*	1.968	7.131***	2.035	3.227*	4.090*	2.986	7.183***	0.171	1.963	4.348*	5.543**
Pairwise comparison		a > c** b > c*		a > c*	a > c*		a > c** b > c*			a > c*	a > c**
Night shifts per week											
No	4.100 ± 0.566	3.756 ± 0.712	4.070 ± 0.527	3.909 ± 0.605	4.049 ± 0.546	4.263 ± 0.528	3.954 ± 0.546	3.108 ± 0.982	4.040 ± 0.604	4.031 ± 0.653	3.932 ± 0.429
Yes	4.014 ± 0.574	3.709 ± 0.726	4.034 ± 0.523	3.824 ± 0.643	4.042 ± 0.545	4.243 ± 0.480	3.961 ± 0.526	3.136 ± 1.053	4.079 ± 0.547	4.000 ± 0.645	3.906 ± 0.428
*t*	1.615	0.69	0.752	1.45	0.125	0.43	−0.14	−0.287	−0.732	0.504	0.658
Whether to teach students											
No	4.066 ± 0.572	3.673 ± 0.748	4.035 ± 0.535	3.845 ± 0.650	4.013 ± 0.562	4.251 ± 0.514	3.943 ± 0.527	3.221 ± 1.052	4.028 ± 0.588	3.991 ± 0.684	3.907 ± 0.428
Yes	4.024 ± 0.573	3.768 ± 0.697	4.055 ± 0.516	3.858 ± 0.617	4.071 ± 0.530	4.248 ± 0.482	3.971 ± 0.537	3.046 ± 1.004	4.097 ± 0.548	4.027 ± 0.616	3.921 ± 0.429
*t*	0.818	**−**1.485	**−**0.417	**−**0.226	**−**1.206	0.072	**−**0.595	1.914	**−**1.371	**−**0.633	**−**0.386

Abbreviation: PSC, patient safety culture.

* *p* < 0.05

** *p* < 0.01

*** *p* < 0.001.

Table 7 shows the comparison of PSC scores by PSC‐related characteristics. No significant differences were found based on levels of direct patient contact. However, respondents from departments without reported safety incidents scored significantly higher on the dimensions “Teamwork,” “Staffing,” “Learning,” “Communication Openness,” and “Management Support” than those from departments with reported incidents. Respondents familiar with PSC scored higher on the dimensions “Teamwork,” “Staffing,” “Learning,” “Error Response,” “Leader Support,” “Error Communication,” “Management Support,” “Handoffs,” and overall PSC than those with moderate familiarity. Those who had participated in PSC training in the last year scored significantly higher on the dimensions “Teamwork,” “Learning,” “Management Support,” and overall PSC than those without such training.

**Table 7:** Comparison of PSC scores by PSC‐related characteristics.

Variables	Teamwork	Staffing	Learning	Error response	Leader support	Error communication	Communication openness	Event reporting	Management support	Handoffs	Total
Direct contact with patients											
Yes	4.045 ± 0.576	3.730 ± 0.726	4.052 ± 0.525	3.853 ± 0.632	4.046 ± 0.542	4.251 ± 0.501	3.959 ± 0.535	3.127 ± 1.033	4.068 ± 0.564	4.013 ± 0.648	3.916 ± 0.428
No	4.000 ± 0.492	3.595 ± 0.605	3.889 ± 0.498	3.826 ± 0.637	4.000 ± 0.611	4.217 ± 0.384	3.957 ± 0.486	3.105 ± 0.951	4.014 ± 0.631	3.955 ± 0.645	3.885 ± 0.433
*t*	0.369	0.840	1.402	0.203	0.399	0.319	0.017	0.092	0.445	0.415	0.348
Occurrence of PSC in the department											
Yes	3.704 ± 0.562	3.487 ± 0.619	3.825 ± 0.489	3.756 ± 0.637	4.008 ± 0.526	4.242 ± 0.465	3.790 ± 0.496	3.385 ± 0.870	3.800 ± 0.635	3.908 ± 0.658	3.792 ± 0.433
No	4.071 ± 0.565	3.745 ± 0.726	4.064 ± 0.523	3.860 ± 0.631	4.047 ± 0.547	4.250 ± 0.500	3.972 ± 0.533	3.105 ± 1.039	4.088 ± 0.556	4.019 ± 0.646	3.925 ± 0.426
*t*	3.952***	2.174*	2.791**	1.000	0.435	0.106	2.095*	−1.899	2.781**	1.040	1.900
Familiarity with PSC											
Unfamiliar^a^	4.000 ± 0.527	3.516 ± 0.738	4.048 ± 0.519	3.667 ± 0.722	3.817 ± 0.488	4.206 ± 0.532	3.849 ± 0.625	3.143 ± 1.051	4.063 ± 0.712	3.817 ± 0.798	3.816 ± 0.463
Moderately familiar^b^	3.832 ± 0.527	3.506 ± 0.679	3.845 ± 0.474	3.693 ± 0.530	3.892 ± 0.507	4.124 ± 0.406	3.854 ± 0.468	3.048 ± 0.951	3.873 ± 0.524	3.866 ± 0.593	3.755 ± 0.371
Familiar^c^	4.096 ± 0.574	3.789 ± 0.719	4.094 ± 0.526	3.900 ± 0.642	4.093 ± 0.548	4.282 ± 0.510	3.989 ± 0.539	3.145 ± 1.048	4.112 ± 0.560	4.055 ± 0.646	3.958 ± 0.430
*F*	8.663***	7.078***	9.069***	6.128**	7.396***	5.402**	2.967	0.336	7.155***	4.325*	9.738***
Pairwise comparison	c > b***	c > b**	c > b***	c > b**	c > b**	c > b**			c > b**	c > b*	c > b***
Participation in patient safety training in the past year											
Yes	4.064 ± 0.571	3.742 ± 0.709	4.067 ± 0.515	3.867 ± 0.627	4.057 ± 0.538	4.256 ± 0.499	3.967 ± 0.529	3.128 ± 1.033	4.080 ± 0.553	4.020 ± 0.649	3.928 ± 0.424
No	3.781 ± 0.527	3.502 ± 0.840	3.776 ± 0.570	3.664 ± 0.663	3.886 ± 0.615	4.167 ± 0.457	3.849 ± 0.568	3.105 ± 0.987	3.886 ± 0.703	3.883 ± 0.615	3.752 ± 0.447
*t*	2.957**	1.716	3.323**	1.908	1.865	1.070	1.321	0.132	2.039*	1.246	2.450*

Abbreviation: PSC, patient safety culture.

* *p* < 0.05

** *p* < 0.01

*** *p* < 0.001.

### 3.4 Factors Independently Associated With PSC

Multiple linear regression analysis was used to further examine the factors associated with PSC. Independent variables included general demographic characteristics, work‐related factors, and PSC‐related variables, with PSC score as the dependent variable. The multivariate linear regression analysis (Table 8) showed that age, monthly income, hospital grade, professional title, and weekly working hours significantly influenced healthcare workers' PSC. Specifically, older workers (31–45 and > 45 years) had lower PSC compared with those aged ≤ 30 years. Higher monthly income (5001–7000 RMB and > 7000 RMB) was associated with increased PSC, whereas working in tertiary hospitals and longer weekly hours (over 50 h) were linked to reduced PSC. Conversely, holding a senior professional title was associated with higher PSC.

**Table 8:** Multivariate linear regression analysis of PSC.

Independent variables	Coefficient	Standard error	*t*	*p*
Gender (Reference group: Male)				
Female	0.161	0.091	1.77	0.077
Age (Reference group: ≤ 30)				
31–45	**−**0.147	0.062	**−**2.38	0.018
> 45	**−**0.245	0.095	**−**2.58	0.010
Education level (Reference group: Associate degree or below)				
Bachelor's degree or above	0.077	0.051	1.49	0.136
Marriage (Reference group: Unmarried*)				
Married	**−**0.003	0.046	**−**0.07	0.945
Monthly income (Reference group: ≤ 5000)				
5001–7000	0.146	0.044	3.36	0.001
> 7000	0.115	0.049	2.34	0.020
Hospital grade (Reference group: Tertiary hospital)				
Secondary hospital	**−**0.188	0.042	**−**4.44	＜0.001
Years of service at current hospital (Reference group: ≤ 10)				
10–15	**−**0.010	0.062	**−**0.16	0.872
> 15	**−**0.062	0.072	**−**0.86	0.388
Professional title (Reference group: Junior level or below)				
Intermediate level	0.071	0.063	1.13	0.260
Senior level	0.253	0.090	2.79	0.005
Employment type (Reference group: Officially employed)				
Contract‐based	0.045	0.051	0.88	0.379
Other	**−**0.087	0.060	**−**1.44	0.150
Department (Reference group: Internal Medicine)				
Surgery	0.068	0.046	1.50	0.135
Others	0.086	0.045	1.89	0.059
Weekly working hours (Reference group: ≤ 40)				
40–50	**−**0.066	0.041	**−**1.58	0.115
> 50	**−**0.150	0.056	**−**2.69	0.007
Night shifts per week (Reference group: no)				
Yes	**−**0.035	0.044	**−**0.80	0.423
Whether to teach students (Reference group: no)				
Yes	**−**0.023	0.041	**−**0.55	0.585
Direct contact with patients (Reference group: no)				
Yes	0.032	0.089	0.36	0.717
Occurrence of PSC in the Department (Reference group: no)				
Yes	**−**0.103	0.068	**−**1.51	0.132
Familiarity with PSC (Reference group: unfamiliar)				
Moderately familiar	**−**0.106	0.099	**−**1.07	0.286
Familiar	0.068	0.093	0.73	0.467
Participation in patient safety training in the past year (Reference group: no)				
Yes	0.107	0.071	1.50	0.135
Constant	3.602	0.323	11.16	＜0.001

*Note:* Model F = 4.219, *p* < 0.001.

Abbreviation: PSC, patient safety culture.

## 4 Discussion

In this study, we used the HSOPSC to assess PSC in TCM institutions. The core dimensions of the HSOPSC, which include teamwork, communication openness, error reporting, and management support, are fundamental to PSC across diverse healthcare systems and cultural contexts. Given the absence of a validated TCM‐specific PSC instrument, use of the HSOPSC is reasonable and scientifically sound. However, we recognize that the unique clinical practices, organizational structures, and cultural philosophies of TCM institutions may not be fully captured by the HSOPSC. Therefore, future research should focus on developing and validating a culturally adapted PSC measurement tool tailored to the specific context of TCM settings.

The findings showed that the average PSC score among healthcare workers in TCM institutions in Zhejiang Province was 3.92, with an overall positive response rate of 81%, indicating a generally high level of PSC. This score is higher than scores reported in previous studies [14, 15]. This may be because all respondents were from TCM institutions, where clinical practice emphasizes a holistic view that focuses on both disease treatment and overall patient well‐being. This perspective may encourage a more comprehensive approach to patient safety management. Among the dimensions, scores were highest on “Error Communication.” This suggests strong departmental information sharing and feedback on safety incidents, reflecting a transparent safety culture. Scores were lower on “Staffing,” consistent with Aboneh [8], indicating a need for improved human resource allocation. Scores were lowest for “Event Reporting,” which aligns with findings from Hamdan [16], possibly owing to concerns about punishment for reporting. This highlights the need to strengthen adverse event reporting systems in TCM institutions.

Differences in PSC were observed according to gender, age, education level, and monthly income. Female healthcare workers demonstrated significantly higher PSC than male workers, consistent with previous studies [17]. This may be because women have stronger empathetic abilities and place greater emphasis on patient safety. The predominance of female respondents may have partly contributed to the higher overall PSC scores observed compared with previous studies [14, 15]. Younger healthcare workers showed higher PSC, with age identified as an independent associated factor. This may be because younger workers have limited work experience and an overly optimistic perception of the healthcare system. However, other studies [18] have reported higher PSC among older workers, suggesting that the relationship between age and PSC remains inconclusive. Healthcare workers with a bachelor's degree or higher showed significantly higher PSC, consistent with Moussavi [19]. This is likely because they have received more systematic training and have greater safety management responsibilities. Conversely, a Brazilian study [20] reported a negative correlation between education level and PSC, suggesting that higher‐educated staff may show more critical awareness of organizational risks. Monthly income was also identified as an independent associated factor, with workers earning more than 5000 CNY reporting higher PSC. This finding is consistent with Zhao [21] and could be attributed to greater job satisfaction and increased motivation to prioritize patient safety.

Significant differences in PSC were observed based on hospital grade, professional title, employment type, department, and weekly working hours. Healthcare workers in tertiary hospitals reported higher PSC than those in secondary hospitals, and hospital grade was an independent associated factor. This finding is consistent with findings from Alswat [22] and may reflect greater investment in teamwork, managerial support, and safety culture initiatives in tertiary hospitals. Professional title was also an independent associated factor, with senior staff showing higher PSC, likely reflecting their extensive clinical experience and deeper understanding of safety principles [16]. Contract‐based workers demonstrated higher PSC on the “Event Reporting” dimension, possibly because the temporary nature of their employment allowed a more objective view of the work environment, promoting greater transparency in reporting. Internal medicine practitioners showed lower PSC, consistent with previous studies [23]. This may be attributed to the complexity of managing chronic or severe conditions, which often requires intensive multitasking and long‐term multidisciplinary collaboration. These factors may increase the risk of communication errors, particularly during patient handoffs. Additionally, respondents working fewer than 40 h per week reported higher PSC than those exceeding 50 h. This is in line with previous findings [24] and suggests that long working hours may increase stress and fatigue, thereby negatively affecting PSC.

Significant differences in PSC were observed among healthcare workers based on their experiences with patient safety incidents, familiarity with PSC, and participation in PSC training. Respondents from departments without reported patient safety incidents showed significantly higher PSC. This may reflect a more proactive safety culture that prevents incidents from occurring. Alternatively, workers in departments that have had safety events may have lower morale, more stress, or feel that the atmosphere is punitive, which could negatively affect PSC perceptions. Although such incidents could lead to more training, improvements in safety culture may not be immediate. Further research is needed to clarify this relationship. Healthcare workers who had participated in PSC training in the last year had higher PSC. Similarly, those familiar with PSC reported higher PSC. These findings highlight the importance of strengthening PSC training to increase healthcare workers' understanding and implementation of patient safety practices.

The present findings could help to inform scientific recommendations to improve PSC among healthcare workers in TCM institutions. Optimizing human resource allocation is essential to alleviating the workload of healthcare workers, reducing stress, and improving their efficiency. Refining adverse event reporting mechanisms and fostering a no‐blame culture are important to encourage transparency and proactive reporting of safety issues. Strengthening PSC training through stratified and targeted education tailored to different professional roles could increase healthcare workers' understanding and implementation of safety practices. Improving information transmission and handover processes is equally important to ensure the accuracy and completeness of medical information during care transitions. Collectively, improving these measures could help to create a more robust and sustainable PSC within TCM institutions.

The strengths of this study are as follows. First, although TCM institutions play an important role in China's healthcare system, empirical research on PSC in these settings remains scarce. This study provides much‐needed evidence on the current status and associated factors of PSC in TCM institutions, thereby contributing to the academic literature and offering a valuable reference for future studies. Second, by analyzing PSC from three dimensions—demographic characteristics, work‐related characteristics, and PSC‐related characteristics—this study provides a more grounded and comprehensive understanding of the factors associated with PSC in TCM institutions. This approach helped to identify key areas for improvement and offered practical references for increasing PSC in similar healthcare settings.

The limitations of this study are as follows. First, although the required sample size was met, the study focused only on TCM institutions in Zhejiang Province, and the generality of the research results requires further confirmation. Future studies should use larger samples to include TCM institutions in multiple regions to improve sample representativeness. Second, the internationally recognized HSOPSC scale was used; however, the specific characteristics of PSC in TCM institutions, such as the risks associated with acupuncture and moxibustion treatments, mean that the scale may not fully reflect the PSC in TCM institutions. Additional research is needed to develop a PSC scale that takes into account the typical characteristics of TCM facilities and is tailored to TCM institutions.

## 5 Conclusion

In this study, the HSOPSC was used to measure the PSC in TCM institutions and explore its associated factors to generate scientific recommendations for improving the PSC in TCM institutions. This study adds to relevant studies on PSC in TCM institutions. The findings showed that the overall PSC in TCM institutions in Zhejiang Province is at a relatively high level. However, the scores for “B Staffing and Work Pace” and “H Reporting Patient Safety Events” were comparatively low. PSC is associated with various factors, including gender, age, education level, monthly income, hospital grade, professional title, employment type, department, weekly working hours, experience with patient safety incidents, familiarity with PSC, and participation in PSC training. Among these, age, monthly income, hospital grade, professional title, and weekly working hours were identified as independent associated factors.

## Author Contributions

**Liujin Zhang:** methodology (lead), software (lead), data curation (lead), writing – original draft (lead), formal analysis (Lead). **Chen Wei:** validation (Lead), investigation (lead), visualization (lead). **Yuan Le:** resources (lead), writing – review and editing (lead). **Fuqiang Chen:** conceptualization (equal), supervision (equal), project administration (equal). **Tao Yang:** conceptualization (equal), supervision, project administration (equal). **Zhiwei Leng:** conceptualization (lead), supervision (lead), project administration (lead), funding acquisition (lead).

## Ethics Statement

According to the National Health Commission of the People's Republic of China's “Measures for Ethical Review of Life Science and Medical Research Involving Humans” document, the use of anonymized information data or biological samples for research can be exempted from ethical review on the premise that the use of human information data or biological samples does not cause harm to the human body, and does not involve sensitive personal information or commercial interests.

## Consent

At the beginning of the questionnaire, participants were informed of the purpose and content of the study, as well as the confidentiality of their responses. The informed consent of the respondents was obtained for this study, and the data was anonymized when the data was analyzed, complying with the exemption from ethical review.

## Conflicts of Interest

The authors declare no conflicts of interest.

## Supporting information


**Supporting Information S1:** Questionnaire on Patient Safety Culture in Traditional Chinese Medicine Institutions.

## Data Availability

Data will be made available on request.
