# Supplementary material for: Status and Factors Associated With Patient Safety Culture in Traditional Chinese Medicine Institutions: A Cross‐Sectional Study
Source: Health Care Sci. 2025 Aug 12;4(5):328–39. doi: 10.1002/hcs2.70029 (PMC12574425; doi:10.1002/hcs2.70029)
Supplement: Supplementary file 1 — Supporting Information S1: Questionnaire on Patient Safety Culture in Traditional Chinese Medicine Institutions. [file HCS2-4-328-s001.docx]

**Questionnaire on Patient Safety Culture in Traditional Chinese Medicine Institutions**

**Dear respondent,**

Hello! We are researchers from the Institute of Clinical Medicine of Peking Union Medical College Hospital, and we would like to invite you to participate in the research investigation of "Patient Safety Culture in traditional Chinese medicine institutions". The purpose of this study is to understand the current situation and existing problems of patient safety culture in traditional Chinese medicine institutions, so as to put forward suggestions to improve patient safety culture. This study adopts anonymous survey, the research time is about 15 minutes, any information involved in the research process and the later research results will only be used for scientific research, we are responsible for the information provided by you strictly confidential.

Thank you again for your participation and support, your comments are vital to continuously improve the patient safety culture in traditional Chinese medicine institutions! I wish you good health and success in your work!

Thank you very much!

Institute of Clinical Medicine, Peking Union Medical College Hospital

**The first part: personal basic situation**

| **Number** | **Item** | **Option** |
| --- | --- | --- |
| 1.1 | Gender | □Male □Female |
| 1.2 | Age | ______Years |
| 1.3 | Education Level | □High school/technical school/technical secondary school and below □Junior college □undergraduate □Master □Doctor |
| 1.4 | Marriage | □Unmarried □Married □Divorced □Widowed |
| 1.5 | Monthly Income | After Tax yuan |
| 1.6 | Professional Title | □Ungraded □Junior level □Intermediate level □Associate senior □Senior |
| 1.7 | Employment Type | □Officially Employed □Contract-Based □Other |
| 1.8 | Hospital Grade | □Tertiary Hospital □Secondary Hospital |
| 1.9 | Department | □Internal Medicine □Surgery □Other |
| 1.10 | In the past year, have there been any patient safety incidents (patient harm caused by medical practices, as opposed to the natural outcome of disease) in your department? | □No □ frequency（Fill in Arabic numerals） |
| 1.11 | Do you have direct contact with patients at work | □No □Yes |
| 1.12 | Your years of service at current hospital | □≤1 □1~5（including 5） □5~10（including 10） □10~15（including15） □≥15 |
| 1.13 | Weekly working hours | □≤40 □40~50（including 50） □＞50 |
| 1.15 | Night Shifts per Week | frequency |
| 1.16 | Your familiarity with patient safety culture | □Unfamiliar □Moderately Familiar □Familiar |
| 1.17 | Have you received any patient safety training in the past year? | □Yes □No（Skip 1.17.1 and go straight to 1.18） |
| 1.17.1 | The number of times you received patient safety training in the past year | frequency |
| 1.18 | Whether to teach students | □No □Yes |

**Part two: Investigation of patient safety culture**

***Patient safety culture:*** *refers to the shared values, attitudes, and codes of conduct of all employees in a healthcare organization with regard to patient safety.*

| **Items** | **Strongly Disagree** | **Disagree** | **Neither Agree nor Disagree** | **Agree** | **Strongly Agree** | **Does Not Apply or Don’t Know** |
| --- | --- | --- | --- | --- | --- | --- |
| A1 In this unit, we work together as an effective team. |  |  |  |  |  |  |
| A2 In this unit, we have enough staff to handle the workload. |  |  |  |  |  |  |
| A3 Staff in this unit work longer hours than is best for patient care. |  |  |  |  |  |  |
| A4 This unit regularly reviews work processes to determine if changes are needed to improve patient  safety. |  |  |  |  |  |  |
| A5 This unit relies too much on temporary, float, or PRN staff. |  |  |  |  |  |  |
| A6 In this unit, staff feel like their mistakes are held against them. |  |  |  |  |  |  |
| A7 When an event is reported in this unit, it feels like the person is being written up, not the problem. |  |  |  |  |  |  |
| A8 During busy times, staff in this unit help each other. |  |  |  |  |  |  |
| A9 There is a problem with disrespectful behavior by those working in this unit. |  |  |  |  |  |  |
| A10 When staff make errors, this unit focuses on learning rather than blaming individuals. |  |  |  |  |  |  |
| A11 The work pace in this unit is so rushed that it negatively affects patient safety. |  |  |  |  |  |  |
| A12 In this unit, changes to improve patient safety are evaluated to see how well they worked. |  |  |  |  |  |  |
| A13 In this unit, there is a lack of support for staff involved in patient safety errors. |  |  |  |  |  |  |
| A14 This unit lets the same patient safety problems keep happening. |  |  |  |  |  |  |
| B1 My supervisor, manager, or clinical leader seriously considers staff suggestions for improving patient  safety. |  |  |  |  |  |  |
| B2 My supervisor, manager, or clinical leader wants us to work faster during busy times, even if it means  taking shortcuts. |  |  |  |  |  |  |
| B3 My supervisor, manager, or clinical leader takes action to address patient safety concerns that are  brought to their attention. |  |  |  |  |  |  |
|  | **Never** | **Rarely** | **Sometimes** | **Most of the time** | **Always** | **Does Not Apply or Don’t Know** |
| C1 We are informed about errors that happen in this unit. |  |  |  |  |  |  |
| C2 When errors happen in this unit, we discuss ways to prevent them from happening again. |  |  |  |  |  |  |
| C3 In this unit, we are informed about changes that are made based on event reports. |  |  |  |  |  |  |
| C4 In this unit, staff speak up if they see something that may negatively affect patient care. |  |  |  |  |  |  |
| C5 When staff in this unit see someone with more authority doing something unsafe for patients, they  speak up. |  |  |  |  |  |  |
| C6 When staff in this unit speak up, those with more authority are open to their patient safety concerns. |  |  |  |  |  |  |
| C7 In this unit, staff are afraid to ask questions when something does not seem right. |  |  |  |  |  |  |
| D1 When a mistake is caught and corrected before reaching the patient, how often is this reported? |  |  |  |  |  |  |
| D2 When a mistake reaches the patient and could have harmed the patient, but did not, how often is this  reported? |  |  |  |  |  |  |
|  | **Strongly Disagree** | **Disagree** | **Neither Agree nor Disagree** | **Agree** | **Strongly Agree** | **Does Not Apply or Don’t Know** |
| F1 The actions of hospital management show that patient safety is a top priority. |  |  |  |  |  |  |
| F2 Hospital management provides adequate resources to improve patient safety. |  |  |  |  |  |  |
| F3 Hospital management seems interested in patient safety only after an adverse event happens. |  |  |  |  |  |  |
| F4 When transferring patients from one unit to another, important information is often left out. |  |  |  |  |  |  |
| F5 During shift changes, important patient care information is often left out. |  |  |  |  |  |  |
| F6 During shift changes, there is adequate time to exchange all key patient care information. |  |  |  |  |  |  |

**Hospital Survey on Patient Safety Culture Scale**

| **Dimension** | **Items** |
| --- | --- |
| A Teamwork | A1 In this unit, we work together as an effective team. |
|  | A8 During busy times, staff in this unit help each other. |
|  | A9 There is a problem with disrespectful behavior by those working in this unit. |
| B Staffing and Work Pace | A2 In this unit, we have enough staff to handle the workload. |
|  | A3 Staff in this unit work longer hours than is best for patient care. |
|  | A5 This unit relies too much on temporary, float, or PRN staff. |
|  | A11 The work pace in this unit is so rushed that it negatively affects patient safety. |
| C Organizational Learning—Continuous Improvement | A4 This unit regularly reviews work processes to determine if changes are needed to improve patient safety. |
|  | A12 In this unit, changes to improve patient safety are evaluated to see how well they worked. |
|  | A14 This unit lets the same patient safety problems keep happening. |
| D Response to Error | A6 In this unit, staff feel like their mistakes are held against them. |
|  | A7 When an event is reported in this unit, it feels like the person is being written up, not the problem. |
|  | A10 When staff make errors, this unit focuses on learning rather than blaming individuals. |
|  | A13 In this unit, there is a lack of support for staff involved in patient safety errors. |
| E Supervisor, Manager, or Clinical Leader Support for Patient Safety | B1 My supervisor, manager, or clinical leader seriously considers staff suggestions for improving patient safety. |
|  | B2 My supervisor, manager, or clinical leader wants us to work faster during busy times, even if it means taking shortcuts. |
|  | B3 My supervisor, manager, or clinical leader takes action to address patient safety concerns that are  brought to their attention. |
| F Communication About Error | C1 We are informed about errors that happen in this unit. |
|  | C2 When errors happen in this unit, we discuss ways to prevent them from happening again. |
|  | C3 In this unit, we are informed about changes that are made based on event reports. |
| G Communication Openness | C4 In this unit, staff speak up if they see something that may negatively affect patient care. |
|  | C5 When staff in this unit see someone with more authority doing something unsafe for patients, they speak up. |
|  | C6 When staff in this unit speak up, those with more authority are open to their patient safety concerns. |
|  | C7 In this unit, staff are afraid to ask questions when something does not seem right. |
| H Reporting Patient Safety Events | D1 When a mistake is caught and corrected before reaching the patient, how often is this reported? |
|  | D2 When a mistake reaches the patient and could have harmed the patient, but did not, how often is this reported? |
| I Hospital Management Support for Patient Safety | F1 The actions of hospital management show that patient safety is a top priority. |
|  | F2 Hospital management provides adequate resources to improve patient safety. |
|  | F3 Hospital management seems interested in patient safety only after an adverse event happens. |
| J Handoffs and Information Exchange | F4 When transferring patients from one unit to another, important information is often left out. |
|  | F5 During shift changes, important patient care information is often left out. |
|  | F6 During shift changes, there is adequate time to exchange all key patient care information. |
